# Exploring the lower thermal limits for development of the human malaria parasite, *Plasmodium falciparum*

**DOI:** 10.1098/rsbl.2019.0275

**Published:** 2019-06-26

**Authors:** Jessica L. Waite, Eunho Suh, Penelope A. Lynch, Matthew B. Thomas

**Affiliations:** 1 Center for Infectious Disease Dynamics and Department of Entomology, The Pennsylvania State University, University Park, PA 16802, USA; 2 College of Life and Environmental Sciences, University of Exeter, Penryn Campus, Cornwall TR10 9FE, UK

**Keywords:** pathogen, extrinsic incubation period, climate change, parasite infection, highlands, malaria risk

## Abstract

The rate of malaria transmission is strongly determined by parasite development time in the mosquito, known as the extrinsic incubation period (EIP), since the quicker parasites develop, the greater the chance that the vector will survive long enough for the parasite to complete development and be transmitted. EIP is known to be temperature-dependent but this relationship is surprisingly poorly characterized. There is a single degree-day model for EIP of *Plasmodium falciparum* that derives from a limited number of poorly controlled studies conducted almost a century ago. Here, we show that the established degree-day model greatly underestimates the rate of development of *P. falciparum* in both *Anopheles stephensi* and *An. gambiae* mosquitoes at temperatures in the range of 17–20°C. We also show that realistic daily temperature fluctuation further speeds parasite development. These novel results challenge one of the longest standing models in malaria biology and have potentially important implications for understanding the impacts of future climate change.

## 1. Introduction

The transmission of vector-borne diseases is strongly influenced by environmental temperature [1,2]. For this reason, there is considerable interest in the possible effects of climate change on the dynamics and distribution of diseases such as malaria (e.g. [3–7]). One of the key temperature dependencies in malaria transmission is the extrinsic incubation period (EIP; also defined as the duration of sporogony), which describes the time it takes following an infectious blood meal for parasites to develop within a mosquito and become transmissible [8].

Most mechanistic models of *Plasmodium falciparum* transmission base estimates of EIP on the degree-day model of Detinova [9], which was founded on work by Moshkovsky [10], Moshkovsky & Rashina [11] and Nikolaev [12] among others:

1.1 $$\mathrm{EIP}(\mathrm{in}\;\mathrm{days})=\frac{111}{\left(T^{\circ }C\text{–}16^{\circ }C\right)}.$$

Degree-day models provide a measure of physiological time and are widely applied in many areas of biology to characterize the influence of temperature on growth rates and phenology. Degree-day models assume a linear relationship between growth rate and temperature, that there is a minimum temperature threshold below which growth rate is zero, and that a fixed number of heat units (degree-days) need be accumulated to complete development once temperature is above the zero threshold (i.e. number of days to complete development multiplied by the effective temperature). In the Detinova model, 111 is the cumulative number of degree-days required for the parasite to complete development, and the effective temperature is the difference between the average ambient environmental temperature, *T*, and a lower temperature threshold of 16°C. However, in spite of widespread use for over 50 years, the Detinova degree-day model is poorly validated (reviewed in [8]). For example, the model was parametrized with limited data from a single study conducted in the 1930s using the Eurasian vector, *Anopheles maculipennis* [12]. This work provided no empirical measurements of EIP below 20–21°C. Similar historic studies either lacked temperature control, adequate sampling, or did not use *P. falciparum* parasites (e.g. [13–15]). To date, virtually no published studies have measured EIP at cooler temperatures, or confirmed the lower developmental threshold. Further, the model is based on constant temperatures, yet temperatures in the field exhibit diurnal fluctuation, which could affect parasite development [16].

Here we use *An. stephensi* and *An. gambiae* mosquitoes to determine the lower temperature threshold of *P. falciparum*, evaluate EIP at temperatures below 20°C, and examine whether the degree-day model is robust to daily temperature variation.

## 2. Material and methods

### (a) Experimental treatments

Mosquitoes were reared at 27°C following standard protocols [17]. *Plasmodium falciparum* (NF54) parasite cultures were either provided by the Johns Hopkins Malaria Institute Core Facility, or produced in our laboratory following protocols described in [17]. In all cases, gametocyte cultures reached approximately 2–4% mature gametocytaemia and were between 14 and 17 days post gametocyte induction when cultures were fed to 3–5 day old mosquitoes. After 20 min, blood-fed females were moved to temperature-controlled incubators, set at 80 ± 5% RH. Mosquitoes were sampled over time with midguts and salivary glands dissected to estimate oocyst and sporozoite infection (sampling intervals given in electronic supplementary material, table S1).

For *An. stephensi* (Liston), a dominant malaria vector in India, Asia and parts of the Middle East [18], we examined constant temperatures of 16, 17, 18 and 20°C, and fluctuating temperature regimes of 14 ± 5, 16 ± 5 and 18 ± 5°C. For *An. gambiae* (NIH G3), the primary vector in sub-Saharan Africa [19], we examined temperatures of 17, 19 and 19 ± 5°C. The daily fluctuations followed a previously described temperature model [3,16]. Diurnal temperature ranges (DTR) of 5–20°C are common across many malaria transmission settings [3,16,20], and so a DTR of 10°C is a representative intermediate value. For each temperature, we had one to six biological replicates using separate infectious feeds, with at least 150 mosquitoes per infectious feed (see electronic supplementary material, table S1 for details of replicate numbers and sample sizes). For each feed, we included a control set of mosquitoes maintained at the regular insectary temperature of 27°C and these were dissected for oocysts to confirm the viability of the infectious feeds.

### (b) Mosquito survival curves at constant and fluctuating temperatures

How EIP affects malaria transmission depends in part on mosquito survival; the absolute duration of EIP does not matter so much as what proportion of mosquitoes survive the EIP to become infectious [17]. Therefore, to parallel the 18°C infection studies, we generated survival curves for adult *An. stephensi.* Mosquitoes 3–5 days old, reared in identical conditions to those in the infection studies, were provided a blood meal and then placed into incubators at either constant 18°C or 18 ± 5°C. Mosquitoes were housed in cups in groups of 10, with a total of 200 mosquitoes per treatment. Cups were examined daily until the last mosquito died.

## 3. Results

The results of the infection experiments are summarized in table 1 (details of sampling and parasite measures are given in electronic supplementary material, table S1).

**Table 1.** Summary of *P. falciparum* infection across a range of temperatures, showing the number of replicate infectious feeds, the number of mosquitoes dissected to examine for oocysts and sporozoites, the day post-blood-feed when sporozoites were first observed in mosquito salivary glands, and the equivalent EIP predicted by the Detinova degree-day model [9].

mosquito species and temperature regime (°C)	no. of replicate infectious feeds	dissection days post feed (and total no. mosquitoes dissected for oocysts, and sporozoites)	presence of oocysts (and maximum prevalence on a given day)	presence of sporozoites (and maximum prevalence on a given day)	day sporozoites first detected in salivary glands	EIP (days) predicted by degree-day model
*An. stephensi*						
16 ± 0	3	40–62 (50, 92)	no	no	n.a.	infinite
17 ± 0	2	34–60 (64, 273)	yes (55%)	yes (57%)	38	111
18 ± 0	3	16–62 (402, 322)	yes (75%)	yes (73%)	33	56
20 ± 0	1	11–39 (148, 114)	yes (80%)	yes (78%)	26	28
14 ± 5	3	17–60 (144, 217)	yes (3%)	no	n.a.	infinite
16 ± 5	6	16–55 (362, 377)	yes (5%)	yes (5%)	30	infinite
18 ± 5	2	15–62 (286, 228)	yes (79%)	yes (85%)	27	56
*An. gambiae*						
17 ± 0	2	34–50 (107, 273)	yes (13%)	yes (3%)	43	111
19 ± 0	2	23–41 (52, 383)	yes (40%)	yes (23%)	29	37
19 ± 5	2	18–40 (80, 491)	yes (50%)	yes (33%)	25	37

We found no evidence for oocyst or sporozoite infection at constant 16°C over three replicate feeds. Oocyst prevalence in mosquitoes maintained at 27°C (controls) ranged from 82 to 100%, indicating that these feeds were infectious (electronic supplementary material, table S1). Positive salivary gland infections were detected at constant 17, 18 and 20°C for *An. stephensi*, and 17 and 19°C for *An. gambiae*. Rates of parasite development were greater than predicted by the established degree-day model.

Adding realistic diurnal temperature fluctuation enabled parasites to establish to oocyst stage at 14 and 16°C, and to complete development to sporozoite stage at 16°C in *An. stephensi*, albeit at low levels (table 1). In the 18 and 19°C treatments, temperature variation further shortened EIP for both vector species (table 1).

**Figure 1.**
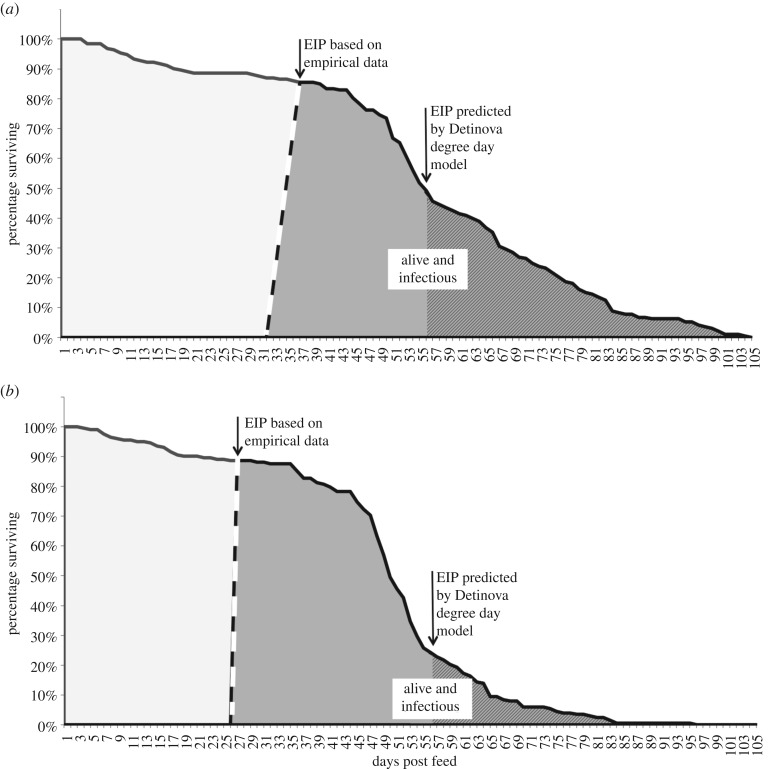
Plot line shows survival of mosquitoes that we assume will become infectious following a parasite-infected blood meal. Areas under the line represent total mosquito days of life, based on empirical data for *An. stephensi* at (*a*) 18°C, and (*b*) 18 ± 5°C. The dashed line bounding the grey area represents estimated EIP, or parasite sporogony, based on observed data at these temperatures. The grey area represents the number of infectious-mosquito-days, which provides a relative measure of force of infection. Within the larger grey area, the hatched shading represents infectious-mosquito-days calculated using the degree-day model of Detinova [9].

Our relatively coarse sampling frequency does not enable us to precisely define EIP (i.e. to definitively capture the day the first individual mosquito becomes infectious). However, by estimating 95% confidence intervals across the replicate infectious feeds we can determine a credible window for EIP, representing the latest day at which no sporozoites were likely to be observed, and the latest day at which maximum sporozoite prevalence was likely to have occurred based on our observations. Our conservative methodology minimized any differences between the observed values and the Detinova values. This approach conceptually follows recent work defining the completion of sporogony as a distribution rather than a single time point [8,17,21]. Using this method, we define a window of sporogony for *An. stephensi* of 31–37 days at 18°C, and 26–27 days at 18 ± 5°C (figure 1*a*,*b*). These empirical estimates are substantially shorter than the EIP of 56 days predicted by the degree-day model. The significance of these shorter EIPs can be illustrated by integrating EIP with the respective mosquito survival (figure 1*a*,*b*). The shaded areas provide a measure of the daily number of infectious mosquitoes alive, or ‘infectious-mosquito-days’, which all else being equal, scales with force of infection [17]. Our empirical data suggest a 2.7-fold increase in force of infection at constant 18°C compared with the degree-day model, and 8.5-fold increase at 18 ± 5°C.

## 4. Discussion

The Detinova model of EIP has been applied extensively for over 50 years, but with little critical evaluation. Our data for *An. stephensi* suggest that 16°C, which is the minimum temperature threshold for *P. falciparum* development assumed in the Detinova model, is a good approximation. While it is possible that there could be vector-by-parasite interactions affecting the threshold in other vector species, studies on the development of *P. falciparum* suggest that exflagellation is constrained at 16°C [22]. Adding realistic diurnal temperature fluctuation at 16°C facilitated infection but only 2/377 *An. stephensi* mosquitoes were positive for sporozoites, so the effect is marginal. A threshold of 16°C is in line with very early empirical work and other parasite kinetics research [22,23], but differs from a number of modelling studies that assume a lower limit of 18°C (reviewed in [24]).

However, the Detinova model dramatically underestimates parasite development rate at temperatures marginally above the lower threshold, and fails to capture the effects of realistic daily temperature variation, which enhances development still further. The Detinova model assumes that 111 cumulative degree-days (heat units) are required to complete sporogony once temperature is above the lower threshold. The current study indicates that the required number of degree-days is not a constant 111 days, but varies nonlinearly with temperature. Based on our observed approximations of EIP (table 1) and the established lower developmental threshold of 16°C, we use the degree-day equation (equation (1.1)) to calculate that sporogony takes as few as 38, 66 and 104 cumulative degree-days at 17, 18 and 20°C, respectively, for *An. stephensi*. For *An. gambiae* we estimate 43 and 87 cumulative degree-days at 17 and 19°C, respectively. Note that these figures are not the actual estimates of EIP and so do not mean that EIP is shorter in absolute terms at cooler temperatures. Rather, our data suggest that at temperatures below 21°C it takes proportionally fewer cumulative ‘heat units’ for parasites to complete sporogony. Moreover, we find that realistic temperature fluctuation can enhance parasite development yet further. These findings are consistent with Jensen's inequality, which predicts that fluctuation around a mean temperature can modify (in this case increase) development rate compared to the same average constant temperature [25]. These are the first empirical data, of which we are aware, to demonstrate that growth rate of human malaria is sensitive to temperature variation, and confirm earlier research conducted using species of rodent malaria [16]. In turn, the faster parasite development yields potentially a much greater force of infection under cooler environmental conditions than predicted using the Detinova model (illustrated in figure 1*a*,*b*).

In contrast to the original empirical work on the EIP of *P. falciparum*, which used a minor malaria vector species from Russia, *An. maculipennis*, our study used key vector species from Africa and Asia. Similar experiments using other primary malaria vectors such as *An. arabiensis*, *An. funestus*, *An. darlingi*, *An. farauti*, etc., would be interesting to determine the generality of our results. More detailed assessment of other strains and species of malaria parasites would also be worthwhile [26]. We acknowledge our mosquito and parasite strains were laboratory-adapted, and there is a need to validate our findings using local mosquito–parasite pairings. Local adaptation in vector and/or parasite populations could change the thermal sensitivity of the vector–parasite interaction [27]. Vector competence and parasite development rate can also be affected by a range of biotic and abiotic factors other than temperature [8,28]. Furthermore, we measured time to invasion of salivary glands by the sporozoites as a proxy for transmission but it might be that this masks some variation in transmission potential between individual sporozoites. At the very least, transmission requires the mosquito to take a blood meal and it is possible for the duration of sporogony and the frequency of feeding (determined in part by the duration of the gonotrophic cycle) to be out of phase [29]. Note, however, these caveats apply equally to the existing Detinova model, yet this single model has been applied to all *P. falciparum*-vector systems worldwide. Similarly, our laboratory-based mosquito survival curves do not necessarily reflect patterns of survival in the field [30,31], yet qualitative differences in force of infection relative to the Detinova model should hold regardless. As such, our results challenge one of the most long-standing models in malaria biology and highlight a need for further laboratory and field studies to examine the thermal ecology of malaria, particularly at the edges of range in areas such as the Kenyan and Ethiopian highlands where the potential impacts of climate change remain controversial [4,5,32,33].

## Supplementary Material

Table S1

## Data accessibility

Data from this study are available as electronic supplementary material, table S1 for all temperatures for mosquitoes dissected for oocysts and sporozoite dissections, sampling intervals, replicates and sample sizes.
